# Laser-Induced Dimeric Photoproducts of Chlorpromazine: LC-MS Identification and Molecular Docking Evidence of Enhanced Anticancer Potential

**DOI:** 10.3390/ijms26146668

**Published:** 2025-07-11

**Authors:** Ana-Maria Udrea, Florin Bilea, Speranta Avram, Angela Staicu

**Affiliations:** 1 National Institute for Lasers, Plasma and Radiation Physics, 409 Atomistilor Str., 077125 Magurele, Romania; ana.udrea@inflpr.ro; 2 Department of Anatomy, Animal Biology, Animal Physiology and Biophysics, Faculty of Biology, University of Bucharest, 91-95 Independentei Sp., 050095 Bucharest, Romania; speranta.avram@bio.unibuc.ro

**Keywords:** chlorpromazine, laser irradiation, molecular docking, HPLC-MS, MS^2^, breast cancer, ADME-Tox predictions

## Abstract

Breast cancer treatments, such as chemotherapy, radiation, and surgery, often face significant limitations, highlighting the need for more effective and targeted therapies. Here, we investigate the potential of 266 nm laser irradiation of chlorpromazine as a novel approach to develop new antitumoral compounds. We identify six chlorpromazine photocompounds with masses in the range of 178–334 u, along with several dimeric compounds with masses between 566 and 600 u, using an HPLC-MS. In silico approaches assess their pharmacokinetic and pharmacodynamic properties while comparing their toxicity with the parent compound. Molecular docking simulations indicate that some photoproducts have a low estimated free energy of binding to cancer-related targets, suggesting enhanced therapeutic potential compared to chlorpromazine. Additionally, ADME-Tox predictions indicate that these photoproducts may have pharmacokinetic and toxicity profiles similar to chlorpromazine. Overall, this study highlights that laser-generated chlorpromazine photoproducts exhibit enhanced biological activity to breast cancer-related targets compared to chlorpromazine while maintaining a similar ADME-Tox profile.

## 1. Introduction

The treatments for breast cancer include surgery, chemotherapy, radiotherapy, endocrine therapy, and molecular targeted therapy. Surgery removes cancerous tissue, while chemotherapy, radiotherapy, and targeted therapies aim to destroy cancer cells throughout the body [1]. MCF-7 is the breast cancer cell line that has produced more data of practical knowledge for patient care than any other breast cancer cell line. MCF-7 is an estrogen (ER)-positive and progesterone (PR)-positive breast cancer cell line used in numerous studies, including those focused on anticancer drugs. Steroid hormone receptors, specifically ER, PR, or both (ER/PR), play a crucial role in prognosis [2]. However, the growth of breast cancer cells is controlled not only by ER and PR but also by plasma membrane-associated growth factor receptors. Two particularly important members of this large family are the epidermal growth factor receptor (EGFR), which is activated by the epidermal growth factor (EGF), and the human epidermal growth factor receptor-2 (HER2), both present in MCF-7 cells [3].

Chlorpromazine (CPZ) is a phenothiazine drug introduced in the 1950s, initially to manage allergic reactions. Today, it remains one of the most widely used and affordable antipsychotics for treating schizophrenia. CPZ has been shown to improve symptoms and overall functioning compared to a placebo. Despite its multiple side effects—such as sedation, movement disorders, Parkinsonism, low blood pressure, and significant weight gain—CPZ is included in the World Health Organisation’s Model List of Essential Medicines as one of five key drugs for managing psychotic disorders [4,5]. In this study, we have explored the use of CPZ, after laser irradiation, as a potential targeted breast cancer treatment for the MCF-7 cell line. Phenothiazines are one of the first lead structures in drug discovery history; they have been studied since their synthesis in 1883, with derivatives being used in the treatment of psychiatric disorders since the 1950s [6,7]. However, their use in cancer treatment is a relatively new development, dating back to the early 2000s [8]. Several studies showed the benefits of phenothiazine-derivatives drugs, including CPZ, in various cancer types [9,10,11,12]. Some of them indicate that CPZ has shown anticancer effects, such as inducing apoptosis and inhibiting DNA synthesis [12,13], while others highlighted CPZ’s inhibitory activity on multiple breast cancer cell lines, including MCF-7, TAMR-1, and MDA-MB-231 [14,15,16]. Nonetheless, to the best of our knowledge, the anticancer effect of CPZ has never been clinically tested as a primary treatment. However, we identified two clinical trials repurposing CPZ as an adjuvant therapeutic agent: one in resected stage III colon cancer and another in newly diagnosed glioblastoma [17,18].

Laser irradiation of CPZ using a 266 nm laser, with an average pulse energy of 6.5 mJ/pulse at 10 Hz and a pulse duration of 6 ns, for varying time intervals, results in the generation of a diverse mixture of compounds, forming a unique ‘cocktail’. Laser-irradiated CPZ has proven to have a high antimicrobial activity compared to a non-irradiated one. Regarding the stability of the laser-irradiated CPZ, Pascu et al. concluded that the solutions remain stable for at least three months [19,20,21,22]. Investigations of pseudotumor have demonstrated the enhanced efficacy of CPZ after 20 min of irradiation at a low concentration [23]. Our previous work studied the laser irradiation effect on CPZ prior administration and showed selective cytotoxicity against breast cancer cells (MCF-7). The study concluded that irradiating CPZ before administration enhanced its efficacy compared to the non-irradiated, suggesting a promising area for targeted therapy development [24].

Alexandru et al. have identified and characterised some of the photoproducts that resulted from the laser irradiation of CPZ [25]. However, this study seeks to further identify the photoproducts. For this, the compounds formed during the irradiation were analysed using High-Performance Liquid Chromatography (HPLC) coupled with tandem mass spectrometry (MS) and their MS^2^ spectra (selected ion fragmentation spectra) were acquired.

Computer-Aided Drug Design (CADD) integrates computational and biological approaches to predict drug–target interactions, pharmacokinetics, and potential toxic or side effects [26]. CADD accelerates the discovery and optimisation of new drug candidates, as well as the repositioning of approved drugs, by using predictive computational models, including those based on artificial intelligence (AI), such as neural networks or deep learning [26,27].

Several drug-like rules, including Lipinsky’s rule of five and Veber’s rule, were applied to the identified photoproducts. These rules serve as guidelines in drug discovery for identifying promising candidates with favourable pharmacokinetic properties [28,29,30]. The Lipinski rule focuses on several physicochemical parameters considered essential for oral bioavailability and pharmacological activity [28]. Similarly, the Veber rule highlights the importance of molecular flexibility and size in optimising oral bioavailability [29].

ADME-Tox is a concept in pharmacology and toxicology that refers to the ADME (Absorption, Distribution, Metabolism, Excretion) of substances within an organism, coupled with their potential toxicological effects (Tox) [31]. To predict the ADME-Tox profile of the identified photoproducts, we chose Deep-PK and ProTox 3.0 [32,33]—the updated versions of ProTox-II and pkCSM—platforms with improved prediction accuracy and broader endpoints relevant to drug-likeness, toxicity, and pharmacokinetics coverage [31,34]. Deep-PK is a deep learning platform that enhances the prediction, analysis, and optimisation of pharmacokinetic (PK) and toxicity properties using Graph Neural Networks and graph-based signatures [31]. ProTox 3.0 is a virtual lab designed to predict the toxicities of small molecules, utilising molecular similarity and machine learning to estimate various toxicity endpoints [34].

This research aims to assess the interaction of the CPZ and its HPLC-MS-identified photoproducts using molecular docking to predict the mechanisms of action as possible targeted anticancer treatments. To achieve this, a comparative analysis will be conducted, aligning phenothiazine derivatives with FDA-approved molecular target drugs for HR-positive or HER2-positive breast cancer types. The drugs under consideration include neratinib (tyrosine kinase inhibitor), as well as letrozole and exemestane (aromatase inhibitors), targeting proteins such as aromatase, FGFR1, estrogen receptor α, EGFR, and HER2 [35].

## 2. Results and Discussions

### 2.1. HPLC-MS Analyses

**Figure 1:**
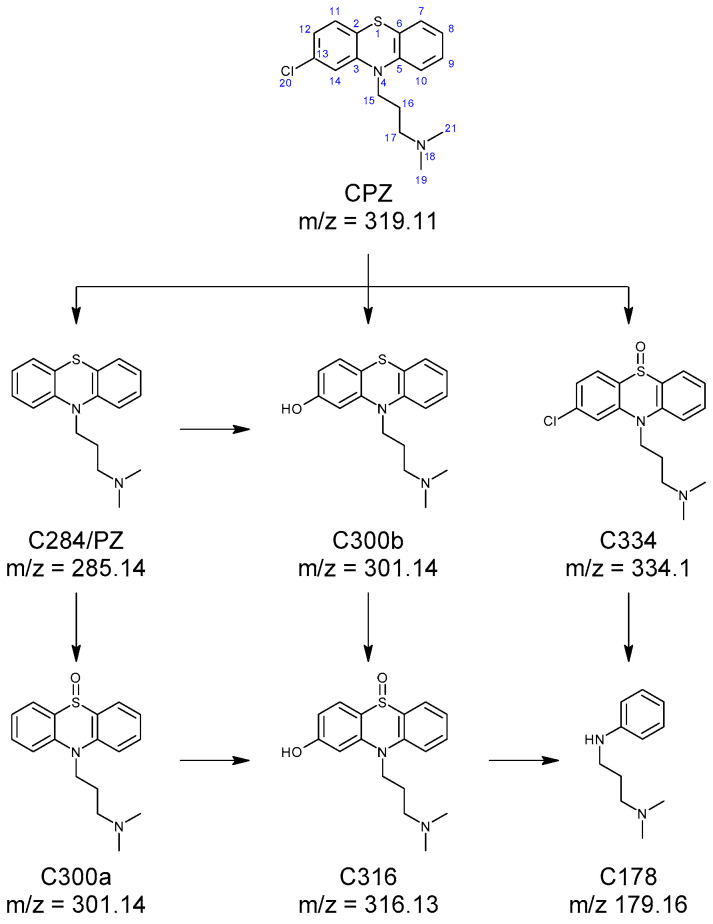
Formation of C178, C284 (promazine), C300a (promazine sulfoxide), C300b (2-hydroxypromazine), C316 (2-hydroxypromazine sulfoxide), and C334 (chlorpromazine sulfoxide) photoproducts of CPZ.

The fragmentation pattern of CPZ (Table S1 Supplementary Materials) has been previously described by Jiménez et al. [36]. Among the MS^2^ fragments, the ions with the mass/charge rations (m/z) of 86.1 and m/z 58.07 are of particular importance, as they correspond to the N,N-dimethylpropanamine moiety from the drug’s structure. When identifying the irradiation products, conservation of the fragments indicates that the change in structure occurs on the phenothiazine moiety. Promazine and promethazine solutions were analysed with the samples. Chromatograms showing the formation of CPZ photoproducts are displayed in Figures S1–S5 Supplementary Materials. The formation of promazine (C284) was confirmed based on the retention time and the typical fragments: m/z 240.09, m/z 212.06, m/z 199.05, m/z 180.08, m/z 86.1, and m/z 58.07 reported in the literature [25,36,37]. Two photoproducts with m/z 301.14 were recorded (C300ab). The mass difference compared to promazine (∆m = 16 u) suggests the presence of an oxygen atom. Both isomers are characterised by the presence of the m/z 86.1 and m/z 58.07 fragments, suggesting that the structure of the phenothiazine moiety was altered. The mass spectrum recorded for C300a has been associated, by Jiménez et al. [36] and Alexandru et al. [25], with promazine sulfoxide, while the one recorded for C300b was attributed to 2-hydroxypromazine [25,36,37]. Another irradiation product observed was C316, having m/z 317.13, which, when compared with promazine, is consistent with the presence of two additional oxygen atoms in the compound’s structure (∆m = 32 u). This photoproduct was identified as 2-hydroxypromazine sulfoxide based on the recorded mass spectrum (Table S1, Supplementary Materials) [25,36]. In the case of C334, the mass difference compared to CPZ (∆m = 16 u) is consistent with the presence of an oxygen atom. Alexandru et al. [25], Jiménez et al. [36], and Trautwein and Kümmerer [37] have attributed a mass spectrum similar to that recorded for C334 to chlorpromazine sulfoxide. The C178 photoproduct has not been previously reported. The presence of the m/z 86.1 and m/z 58.07 fragments in the compound’s mass spectrum suggests the conservation of the N,N-dimethylpropanamine moiety, while the m/z 77.04 fragment can be associated with the presence of a benzene ring. A proposed formation pathway for C284, C300ab, C316, C335, and C178 is shown in Figure 1. These compounds are generated as a direct or indirect result of laser irradiation of the CPZ solution. The direct mechanism implies the homolytic cleavage of covalent bonds such as C-Cl, resulting in the formation of C284, and even cleavage of the C-S and C-N bonds, which could yield C178 [38,39,40]. The indirect mechanism is strongly related to the photolysis of water molecules upon laser irradiation, which yields hydroxyl radicals [41,42]. These radicals can perform dichlorination [43] of CPZ with the formation of C300b or oxidation of the thioether moiety of either CPZ or C284 to sulfoxide (C334 and C334) [44].

During the analysis, a few unexpected products were recorded, having m/z in the range 567–601 (C566ab, C582abcde, C598ab, and C600abc). For these compounds, both [M + H]^+^ and [M + 2H]^2+^ ions were observed, supporting the idea that they represent dimers of CPZ and/or photoproducts. The absence of an obvious monomer in the MS^2^ spectra of the compounds further cements the dimer hypothesis. This type of finding has been previously reported in the case of promethazine exposed to UV light [45] and phenothiazine undergoing electrochemical oxidation [46]. All identified dimers retain the m/z 86.1 and m/z 58.07 fragmentation pattern conserved from CPZ, implying the involvement of the phenothiazine moiety in the dimerisation reaction. C566ab have been identified as promazine dimers. Fragmentation of the [M + 2H]^2+^ shows the simultaneous formation of m/z 482.18 and m/z 86.1, while the m/z 482.18 to m/z 409.09 mass transition (∆m = 73 u) corresponds to the loss of C_4_H_11_N from the second N,N-dimethylpropanamine moiety. Thus, for both compounds, the covalent bond is formed between the phenothiazine moieties, at different positions, which correlates with the presence of the m/z 396.08 fragment, corresponding to a phenothiazine dimer radical cation. In the case of C582abcde, the detected m/z matches C566ab, with an additional oxygen atom, suggesting that the compounds comprise C284 covalently bonded to either C300a or C300b. In the spectra of C582a, the presence of the m/z 300.14 and m/z 284.15 fragments correlate to carbon-centred radicals of C284 and C300ab. For the remaining isomers (C582bcde), the m/z 412 (m/z 396 + 16 µ) fragment corresponds to a phenothiazine dimer radical cation where one of the monomers is either a sulfoxide or an alcohol. Thus, the C582abcde isomers can be differentiated based on the position of the oxygen atom and the position of the bond between the monomers. C598ab are consistent with phenothiazine dimers, with two additional oxygen atoms, either as hydroxyl or sulfoxide groups. The fragmentation of the [M + 2H]^2+^ ion leads to the formation of the m/z 514 and m/z 86.1 ions, accounting for one of the N,N-dimethylpropanamine moieties. For C598a, the m/z 498.23 to m/z 414.09 (∆m = 84 u) accounts for the loss of the second N,N-dimethylpropanamine moiety as C_5_H_10_N^•^. In the case of C598b, the loss of C_5_H_12_N^•^ was observed (m/z 514.17 to m/z 428.07, ∆m = 86 u), suggesting that both compounds result from the formation of the covalent bond between the phenothiazine moieties.

**Figure 2:**
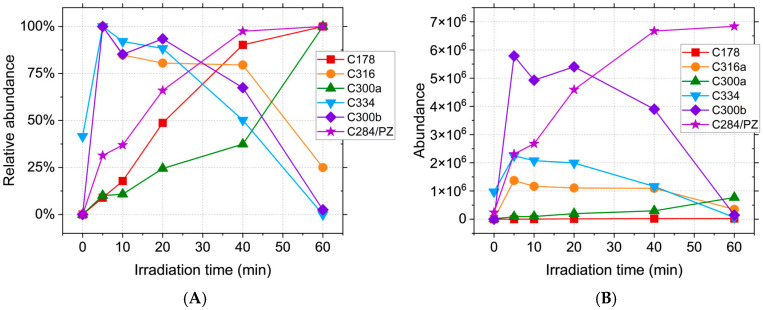
(**A**)**.** Relative abundance and (**B**). abundance of CPZ irradiation products, where relative abundance = recorded abundance/maximum recorded abundance × 100.

**Figure 3:**
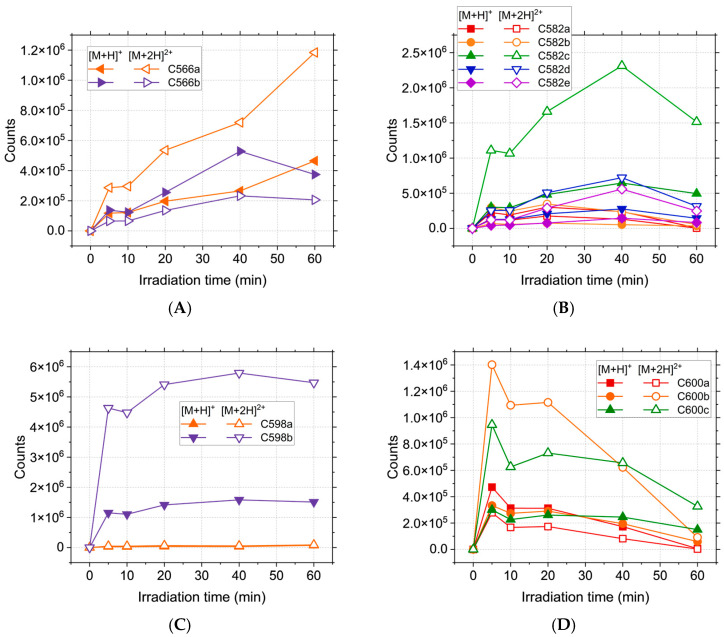
Abundance of high m/z photoproducts of CPZ irradiation over the course of 60 min: (**A**) C566ab; (**B**) C582abcde; (**C**) C598ab; (**D**) C600abc.

The recorded precursor ions for C600abc (m/z 601.22) are consistent with CPZ–promazine dimers. In the case of C600a, this proposed structure is supported by the formation of the m/z 318.12 and m/z 284.13 radical cations upon fragmentation of the [M + 2H]^2+^ ion. For C600bc, fragmentation of the same ion simultaneously forms m/z 516.14 and m/z 86.1, while the m/z 516.14 to m/z 443.05 is consistent with the loss of C_4_H_11_N from the second N,N-dimethylpropanamine moiety, thus showing that the bond between the CPZ and promazine is formed with the participation of the two phenothiazine moieties. Figure 2 and Figure 3 show the abundance of the CPZ photoproducts over the course of laser irradiation (5, 10, 20, 40, 60 min). The relative (Figure 2A) and absolute (Figure 2B) abundance clearly illustrate the time variation in each photoproduct. The reason for this type of representation is that each compound has a different ionisation efficiency depending on its structure [47]. Because of that, the abundance cannot be directly correlated with the concentration in solution. As such, compounds with low ionisation efficiencies would have a low abundance regardless of concentration, thus falsely implying a low concentration in solution. Thus, the abundances of different compounds cannot be directly compared without knowing the ionisation efficiency. In order to bypass this restriction, the relative abundance can be calculated as the measured abundance normalised by the maximum recorded abundance for each compound.

However, the ionisation energy is likely to have little variation for isomers [48], thus yielding a higher probability of correlation with the actual concentration; as such, Figure 3 shows the likely preferential formation of certain compounds of the isomer sets. In this case, the use of the relative abundance would be detrimental to the reality of the results, falsely implying the equal formation of all compounds, which is likely untrue. The C284 compound is formed continuously during irradiation (Figure 2) and reaches a maximum abundance for the longest tested irradiation time (60 min), as is also the case with C178 and C300a. C300b, C316, and C334 reach a maximum at the beginning of the process (5 min), thus implying that the hydroxyl radical reactions take place predominantly for the shortest irradiation times.

While most of these compounds are not present in the initial solution and thus, to some degree, require laser irradiation in order to be formed, the C334 has a high relative abundance even in the absence of treatment, suggesting that the oxidation of CPZ with the formation of chlorpromazine sulfoxide happens spontaneously in the solution. C566ab are steadily formed during irradiation (Figure 3A) up until the 40 min mark, when C566b suffers a decline while C566a reaches a maximum abundance at 60 min.

Of the C582abcde isomers, C582c is the most abundant, followed by C582d and C582e (Figure 3B), and all three have the maximum concentration at 40 min. Of the two C598ab compounds, C598b appears to be preferentially formed during irradiation. Its abundance shows a sharp increase at the beginning of the process, followed by a plateau, implying a constant formation and degradation of this product. As expected, C600abc reach a maximum abundance at the beginning of the irradiation (5 min), followed by a decrease over the course of the treatment. This behaviour can be attributed to the formation of these compounds from two CPZ molecules, of which one undergoes homolytic cleavage of the C-Cl bond [49,50]. The resulting promazine carbon-centred radical can potentially react with CPZ or other photoproducts in order to either form promazine, 2-hydroxypromazine, or one of the dimers containing a promazine moiety [25,51,52]. While the formation of promazine and most dimeric photoproducts are competing reactions, these dimers can also serve as precursors for C284. Homolytic cleavage of all C566, C582, and C600 isomers can generate C284, thus contributing to the total abundance of promazine displayed in Figure 2. Because the maximum abundance of C284 is reached for the longest treatment time, the compounds formed from it should display the same behaviour. This is the case for C300b, but not C300a. While C284 can form both compounds, it only serves as the main precursor for C300b. C300a can also be generated directly from CPZ, the concentration of which is continuously declining during the process, thus impacting the overall abundance. The mass spectra recorded for the photoproducts do not provide enough data in order to properly distinguish isomers with the same m/z and different retention times. Thus, the possible chemical structures have been randomly distributed between these isomers in order to provide a clear view of the results.

### 2.2. Drug-like and ADME-Tox Predictions

In this in silico study, we drew and optimised the molecular structures of CPZ, its identified photoproducts, and the clinically used drugs exemestane, letrozole, and neratinib. Table S2 in the Supplementary Materials presents the SMILES codes and the 2D structures of CPZ and its identified photoproducts using the HPLC-MS analyses.

For the identified photoproducts and CPZ, we used SwissADME webserver to predict several drug-likeness filters, including Lipinski’s Rule of Five [53], Veber (Table 1), Ghose, Egan, and Muegge, the bioavailability score, PAINS and Brenk alerts, lead-likeness violations, and synthetic accessibility (Table S3, Supplementary Materials) [54]. The results are presented in Table 1 and in Table S3, Supplementary Materials.

The SwissADME results indicate that the CPZ photoproducts C178, C284/PZ, C300, C316, and C334 conform with Lipinski’s Rule, Veber, bioavailability criteria, Ghose, and Egan. However, the compounds C566, C582, C598, and C600 do not adhere to Lipinski’s Rule due to their molecular weights exceeding 500 Daltons and MLOGP values greater than 4.15. This suggests that these compounds are unlikely to be orally active, and alternative methods of administration, like parenteral, should be considered in future studies [55,56]. None of the dimers meet the criteria for Ghose, Egan, or Muegge filters; CPZ and C178 also fail Muegge’s filter. The predicted oral bioavailability indicates that the dimers exhibit significantly lower scores (0.17) compared to CPZ (0.55); therefore, alternative administration routes should be considered. The dimeric compounds, C284/PZ and C300a, trigger one PAINS alert (het_thio_666_A), suggesting a potential risk of assay interference that must be considered in future tests [57]. However, in our previous work, we used several biological assays—including the MTS viability assay, LIVE/DEAD staining, LDH release, ROS and nitric oxide production, and F-actin fluorescence staining—to confirm the selective cytotoxicity of laser-irradiated CPZ against breast cancer cells, despite the PAINS alert [24]. Regarding synthetic accessibility, all dimeric compounds are predicted to be more difficult to synthesise than CPZ.

Our ProTox predictions are presented in Table 2 and show that the photoproducts are similar to CPZ regarding toxicity (Table 2).

All the compounds present neurotoxicity and respiratory toxicity; none of them are cardio- or nephrotoxic. Regarding hepatotoxicity, compounds C284, C566, and C600 are predicted to be hepatotoxic according to ProTox 3 predictions, similar to CPZ, while the other photocompounds are predicted to be safe. CPZ and C600 photoproducts are active on the Tumour Suppressor receptor p53. The LD50 of the photoproducts is higher than CPZ, and most of the compounds have a higher or similar toxicity class compared to CPZ (Table 2). To evaluate the ADME-Tox properties of the photoproducts compared with CPZ, we used the Deep-Pk platform. The results show a high similarity between CPZ and its photoproducts, with exceptions in terms of bioavailability, where C582c shows non-bioavailability (Table S4, Supplementary Materials). Regarding the fraction unbound, CPZ has a predicted value of 1.96, lower compared with C566 and C600 compounds. Plasma-protein-binding predictions show that CPZ has a therapeutic index of 44.24, which is lower than 90 and indicates a poor value. Compounds like C566, C582, C589, and C600 present higher therapeutic indexes than CPZ, but still lower than 90. All the photoproducts and CPZ present a high steady-state volume of distribution (VDss) (log VDss > 0.45), indicating that the drug is distributed in tissue rather than plasma. For an anticancer drug, a high VDss is considered an advantage (Table S4, Supplementary Materials). Compounds CPZ, C178, C284/PZ, C300a, C300b, C316, and C334 are inhibitors of Organic Cation Transporter 2 (OCT2), meaning that the photoproducts can interfere with its function and may (i) alter the drug excretion, (ii) affect the pharmacokinetics of other drugs that are substrates of OCT2, (iii) enhance the effectiveness of certain drugs by prolonging their presence in the bloodstream or tissues, and also (iv) have implications for renal function.

All the photoproducts and CPZ are safe in terms of biodegradation. Except for C300b, C316a, and C582e, the photoproducts and CPZ have a high maximum tolerated dose. CPZ and photoproducts C566, C582, C598, and C600 are SR-p53 activators and represent a promising approach in oncology, aiming to leverage the natural cancer-suppressing functions of p53 by reactivating its role in affected cells (Table S4, Supplementary Materials).

### 2.3. Molecular Docking Approach

The molecular docking results, presented in Table 3, indicate that CPZ photoproducts C178, C284, C300a, C300b, C316, and C334 generally exhibit higher Estimated Free Energy of Binding (EFEB) compared to CPZ when interacting with aromatase, ER, FGFR1, EGFR, HER2, PR, and Beclin 1. However, there are exceptions: C334 interacts with aromatase and FGFR1, and C300a interacts with FGFR1, while C300a/b interacts with Beclin 1, all showing lower EFEB values.

Compounds C566, C582, C598, and C600 demonstrate lower EFEB compared to CPZ when binding to aromatase, FGFR1, EGFR, HER2, PR, and Beclin 1 (Table 3).

**Figure 4:**
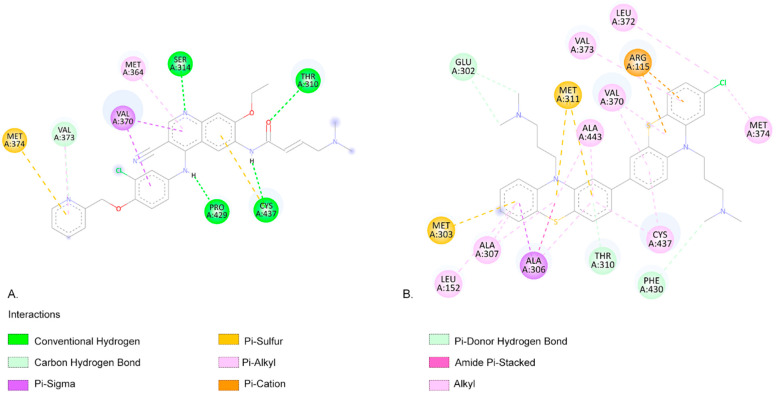
(**A**). The two-dimensional representation of the neratinib structure and the aa residues from its binding site when interacting with the aromatase receptor. (**B**). The two-dimensional structure of C600b and the aa residues from its binding site. Both neratinib and C600b compounds exhibit favourable interactions with the aa residues THR310, VAL370, VAL373, MET374, and CYS437.

A lower EFEB (kcal/mol) indicates a higher probability that the compound will interact with the specific target [58]. According to our predictions, CPZ and most of its identified photoproducts have low predicted binding energies. We compared our results with the medications used in the targeted treatment of breast cancer, exemestane, letrozole, and neratinib, and the results show that photocompounds C566, C582, C598, and C600 have similar or lower EFEB values (Table 3). The lowest EFEB between a photoproduct and a target was obtained when C600b interacts with aromatase (−11.81 kcal/mol), similarly to neratinib. Neratinib and the C600b photoproduct also interact with similar amino acids (aa) (Figure 4). Neratinib forms four conventional hydrogen bond interactions with aromatase aa residues THR310, SER314, PRO429, and CYS437. The C600b compound also binds similarly to the aromatase receptor. Similarly to the results obtained from Hong et al., both neratinib and C600b form favourable interactions with the aa residue THR310 [59].

**Figure 5:**
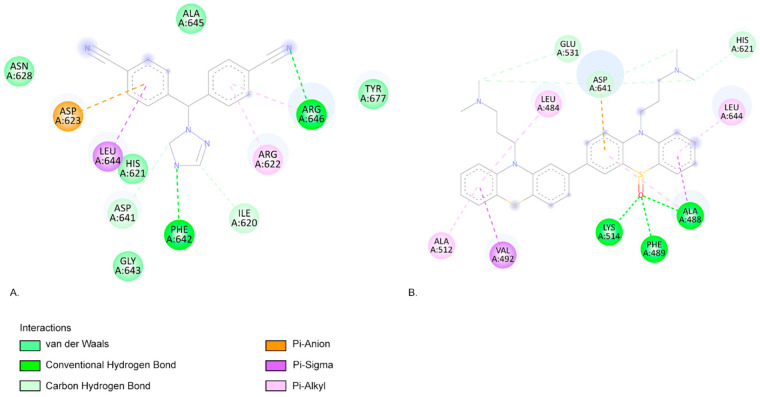
(**A**). The two-dimensional representation of the letrozole structure and the aa residues from its binding site when interacting with the FGFR1. (**B**). The two-dimensional structure of C582e and the aa residues from its binding site. Both letrozole and C582e compounds present favourable interactions with the aa residues ASP641, HIS621, and LEU644.

When interacting with FGFR1, the CPZ-identified photoproduct C582e has the lowest EFEB (−9.81 kcal/mol) according to our results. Among the clinically used compounds we tested, letrozole has the lowest predicted binding energy (−8.79 kcal/mol). Both compounds have favourable interactions with FGFR1 (Figure 5).

Letrozole forms two conventional hydrogen bond interactions with aa residues PHE642 and ARG646. C582e forms three hydrogen bonds with aa residues LYS514, ALA488, and PHE489. The LYS514 aa residue is part of the active site of FGFR1 [60]. Pan et al.’s molecular docking simulations also highlight that ASP641 and PHE 642 are key residues for FGFR1 bioactive inhibitors [60]. Similarly, Ravindranathan et al., in their study on new FGFR1 inhibitors, found that the most effective compound, like C582e in our results, forms hydrogen bonds with LYS514 and PHE489 (Figure 5) [61]. Klein et al. also indicate that aa residues LYS514, ASP641, and ALA488 form the FGFR1-binding site [62].

**Figure 6:**
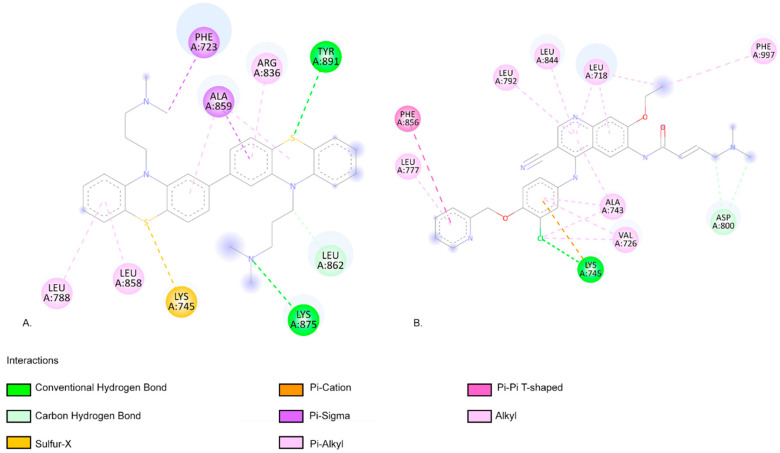
(**A**). The two-dimensional representation of the C566a structure and the aa residues from its binding site when interacting with the EGFR. (**B**). The two-dimensional structure of neratinib and the aa residues from its binding site. Both neratinib and C566a compounds present favourable interactions with the aa residue LYS 45, indicating similar predicted binding sites.

**Figure 7:**
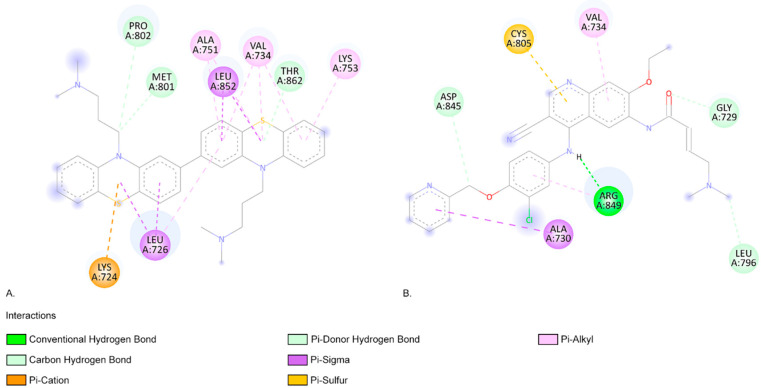
(**A**). The two-dimensional representation of the C566a structure and the aa residues from its binding site when interacting with HER2. (**B**). The two-dimensional structure of neratinib and the aa residues from its binding site. Both neratinib and C566a compounds present favourable interactions with the aa residue VAL 734, indicating similar predicted binding sites.

The C566a photoproduct has the lowest binding affinity when interacting with EGFR (−11.07 kcal/mol) (Figure 6) and HER2 (−10.07 kcal/mol) targets, similar to neratinib (−11.04 kcal/mol and −9.41 kcal/mol, respectively (Figure 7)).

As shown in Figure 6, neratinib forms a hydrogen bond with the aa residue LYS745 of EGFR. The C566a photoproduct forms two conventional hydrogen bonds with the aa residues LYS875 and TYR891. LYS745 is a highly conserved aa residue across all kinases, and its role in phosphotransfer during catalysis is crucial [63]. Additionally, the C566a photoproduct forms a pi–sigma interaction with PHE723 from the p-loop residue of EGFR (Figure 6). Stover et al. reported that aa residues TYR891 and TYR920 are highly phosphorylated in the breast tumour cell line MCF7 [64].

As shown in Figure 7, the HER2 aa residue and ARG 849 aa residue form a conventional hydrogen bond when interacting with neratinib (Figure 7). The photoproduct C566a exhibits positive binding interactions with multiple aa residues within the HER2-binding pocket, including LEU726, VAL734, ALA751, LYS753, MET801, and LEU852 (Figure 7) [65]. In an in silico screening study, Tung et al. also identified favourable interactions between several alkaloids (such as Sanguinarine, Sorafenib, and Tomatidine, which are potential inhibitors of HER2 in cancer treatment) and the aa residues identified in our molecular docking study (Figure 7) [65]. C566a forms a pi–alkyl interaction with LYS753, a residue that engages in hydrophobic contact with the adenine ring of ATP and plays a role in coordinating the ATP catalytic site. This residue is often targeted by inhibitor drugs to block ATP binding [66,67,68]. Additionally, MET801, located in the hinge region of HER2, is known to interact with ATP or other kinase inhibitors [69,70,71]. C566a forms a carbon–hydrogen bond with MET801 (Figure 7).

In this study, using the LC-MS, we identified the photoproducts generated after laser irradiation, and also, using the molecular docking prediction, we evaluated their possible mechanism of action in cancer therapy. The LC-MS analysis shows that, after irradiation, CPZ forms 18 photocompounds, 5 of which are typical of chlorpromazine degradation under laser irradiation. These included compounds that resulted from chlorpromazine dechlorination (promazine, 2-hydroxypromazine) as well as sulfoxides (promazine sulfoxide, 2-hydroxypromazine sulfoxide, chlorpromazine sulfoxide). Moreover, these compounds reacted either with chlorpromazine or with each other in order to form a series of dimeric photoproducts, which have been identified successfully using the MS^2^ spectra of the [M + H]^+^ and [M + 2H]^2+^ cations. The molecular docking simulations indicate that the dimeric compounds have lower binding EFEB values compared to CPZ and the monomeric photocompounds, suggesting a stronger binding affinity. Dimeric compounds C566a and C600b demonstrate particularly strong interactions.

C566a shows a low EFEB across several key targets, including −11.77 kcal/mol with aromatase, −11.07 kcal/mol with EGFR, and −10.07 kcal/mol (Figure 6A) with HER2, indicating a higher binding affinity compared to CPZ, which has EFEB values of −7.64, −8.42, and −7.56 kcal/mol for the same targets. Similarly, C600b binds strongly to aromatase −11.81 kcal/mol and EGFR −10.26 kcal/mol), surpassing CPZ’s binding efficiency. These photocompounds show a promising therapeutic potential due to their higher binding affinity, suggesting they might be more effective in inhibiting breast cancer-related targets than CPZ itself. The lower binding compared with CPZ affinity in target receptors indicates that a lower dose of compounds may be necessary to achieve the same anticancer effect.

The ADME-Tox profile analysis indicates that some CPZ photocompounds exhibit similar or even improved tolerability compared to CPZ. While CPZ is classified as toxicity class 3, photocompounds C178, C300b, C316a, C566a,b, C582a,b,c, and C598b are classified as toxicity class 4. The CPZ LD50 of 125 mg/kg is lower compared with the predicted LD50 of all the photocompounds; for example, the C178 (LD50 of 560 mg/kg) and C582 variants (LD50 up to 408 mg/kg) show improved safety. Moreover, photocompounds C178, C300a,b, C582a-e, and C598a,b, exhibit no activity in targets like drug-induced liver injury (dili) or p53-signalling-related toxicity (sr_p53), indicating potential safety advantages.

## 3. Materials and Methods

### 3.1. Chemicals

Chlorpromazine hydrochloride (CPZ, Sigma-Aldrich Chemie GmbH, Taufkirchen, Germany) was dissolved at a 2 mg/mL concentration in distilled water. The solution was kept at 4 °C and protected from environmental light before and after the irradiation protocol.

### 3.2. Irradiation Protocol

A 2 mL volume of the CPZ solution was irradiated for 5, 10, 20, 40, or 60 min with a 266 nm pulsed beam emitted by a Nd:YAG laser, Continuum (San Jose, CA, USA), (10 Hz, 6 ns FWHM, 6.5 mJ/pulse, 1.08 MW/pulse) according to the protocol detailed in [25,72]. The 266 nm irradiation was selected to match the main UV absorption band of CPZ [21,24].

### 3.3. HPLC-MS

The irradiation products of CPZ were investigated using high-performance liquid chromatography coupled with tandem mass spectrometry (HPLC Agilent Infinity II 1260 (Agilent, Santa Clara, CA, USA) with an Agilent 6530 Quadrupole-Time of Flight detector (Q-TOF)). The separation of compounds in the samples was achieved on a Zorbax Eclipse Plus RP-C18 column (150 × 3.0 mm, 3.5 µm) at 30 °C using a gradient elution method. The mobile phase comprised water with 0.1% formic acid (A) and acetonitrile (B) and was varied as follows: 0 min–10% B → 10 min—50% B → 11 min—50% B → 11.5 min—10% B → 14 min—10% B (flow rate 0.6 mL/min).

The electrospray ionisation source (Dual AJS ESI) parameters were gas—8 L/min, 300 °C; sheath gas—12 L/min, 350 °C; nebuliser—60 psig; capillary voltage—3500 V; nozzle voltage—1000 V; and fragmentor potential—170 V. The mass spectra (MS and MS^2^) were acquired in the positive ionisation mode, in the range 50–1000 m/z (mass/charge), for z = 1 and z = 2, and at a scan rate of 2 spectra/s. Two precursors per cycle were selected for MS^2^ based on abundance, and fragmentation was performed using collision cell potentials of 5, 10, 15, and 20 V. The identification of the molecular formula of each compound was performed based on the isotopic profiles recorded in the Full Scan MS spectra. The mass accuracy was checked for the most abundant monoisotopic ions.

The abundance of the identified photoproducts was attributed to the peak height in the extracted ion chromatograms targeting the corresponding m/z value for each compound. The relative abundance was calculated from the recorded abundances as percentages of the maximum value for each selected m/z value.

### 3.4. Molecular Modelling

#### 3.4.1. Chemical Structure Retrieval and Drawing

The 2D chemical structures of CPZ and its photoproducts were obtained using the SMILES notation from the PubChem database [73,74]. For the photoproducts, Marvin Sketch software version 21.20.0, 2021, [75] was employed to draw and to generate the SMILES codes of the photoproducts’ chemical structures identified following the laser irradiation of CPZ.

#### 3.4.2. Molecular Optimisation

The 3D optimisation of molecular structures was performed using Marvin Sketch software version 21.20.0, 2021, [75]. The molecules were geometry-optimised using the ‘Clean in 3D → Fine Build’ function in MarvinSketch, which automatically generates conformers for molecular fragments using the Dreiding force field, returning the lowest-energy structure [75]. The optimised molecules were saved in a .mol2 file format.

#### 3.4.3. Preparation for Molecular Docking

For compatibility with the molecular docking simulations software Autodock 4.2.6, the optimised structures were converted to pdbqt format. This conversion was facilitated by the use of Open Babel software, version 3.1.1 [76].

### 3.5. Drug-Likeness Evaluation of Photoproducts

We applied several screening criteria, including Lipinski and Veber rules, to assess the drug-likeness of the photoproducts using SwissADME webserver [53,54].

### 3.6. ADME-TOX Predictions

To predict the ADME-Tox profiles of the photoproducts, their SMILES codes were utilised. The analyses were conducted using the Deep-PK platform and ProTox 3.0, which evaluate their pharmacokinetic and toxicological properties [31,34].

### 3.7. Molecular Docking

Autogrid and Autodock software, versions 4.2.6 [77] were used to predict the mechanism of action of CPZ and its photoproducts, regarding the interaction with the following proteins that are targeted by anticancer medication: human oestrogen receptor alpha (ER) (PDB ID: 6VPF) [78], human androgen receptor (AR) (PDB ID: 1E3G) [79], aromatase (PDB ID: 3S7S) [80], basic fibroblast growth factor receptor 1 (FGFR1) (PDB:3GQL) [81], progesterone receptor (PDB ID: 1SQN) [82], and Beclin 1 (PDB ID: 4DDP) [83].

We have used our previously described molecular docking protocol described in [30,58,84]. The grid parameters were set as described in Table 4.

## 4. Conclusions

Laser irradiation at 266 nm changes the CPZ chemical structure, generating 18 photoproducts, including typical degradation products and a series of dimeric compounds. The molecular docking simulations suggest that most of the dimeric photocompounds have lower EFEB values than key targets involved in breast cancer therapy (such as aromatase, ER, FGFR1, EGFR, and HER2) compared to CPZ and its monomeric photocompounds, indicating a potentially more effective cancer treatment at lower doses. The ADME-Tox analysis also suggests improved safety for certain photocompounds, with higher LD50 values and reduced toxicity risks. This highlights that CPZ photoproducts may represent potential candidates for safer, targeted breast cancer therapy. However, future work will focus on the isolation, characterisation, and individual biological tests to assess the identified photoproducts’ anticancer activity.

## 5. Patents

The photoproducts identified and described in this study, along with their predicted inhibitory activity on breast cancer targets, are protected under the Romanian State Office for Inventions and Trademarks (Patent Application Number: A/00409).

## Figures and Tables

**Table 1:** Drug-like predictions of the photoproducts.

Compound	Lipinski Rule of Five Validation	Veber Validation
CPZ	Yes, 1 violation: MLOGP > 4.15	Yes
C178	Yes	Yes
C284/PZ	Yes	Yes
C300a	Yes	Yes
C300b	Yes	Yes
C316a	Yes	Yes
C334	Yes	Yes
C566a	No; 2 violations: MW > 500, MLOGP > 4.15	Yes
C566b	No; 2 violations: MW > 500, MLOGP > 4.15	Yes
C582a	No; 2 violations: MW > 500, MLOGP > 4.15	Yes
C582b	No; 2 violations: MW > 500, MLOGP > 4.15	Yes
C582c	No; 2 violations: MW > 500, MLOGP > 4.15	Yes
C582d	No; 2 violations: MW > 500, MLOGP > 4.15	Yes
C582e	No; 2 violations: MW > 500, MLOGP > 4.15	Yes
C598a	No; 2 violations: MW > 500, MLOGP > 4.15	Yes
C598b	No; 2 violations: MW > 500, MLOGP > 4.15	Yes
C600a	No; 2 violations: MW > 500, MLOGP > 4.15	Yes
C600b	No; 2 violations: MW > 500, MLOGP > 4.15	Yes
C600c	No; 2 violations: MW > 500, MLOGP > 4.15	Yes
exemestrane	Yes	Yes
letrozole	Yes	Yes
neratinib	Yes; 1 violation: MW > 500	No; 1 violation: Rotors > 10

**Table 2:** Hepatotoxicity (dili), neurotoxicity (neuro), nephrotoxicity (nephro), respiratory toxicity (respi), cardiotoxicity (cardio), hosphoprotein (Tumour Suppressor) p53 (sr_p53) activity, Lethal Dose 50 (LD50), and toxicity class of the photoproducts compared with CPZ.

Target	Dili	Neuro	Nephro	Respi	Cardio	sr_p53	LD50 (mg/kg)	Toxicity Class
Compound								
CPZ	Active	Active	Inactive	Active	Inactive	Active	125	3
C178	Inactive	Active	Inactive	Active	Inactive	Inactive	560	4
C284/PZ	Active	Active	Inactive	Active	Inactive	Inactive	210	3
C300a	Inactive	Active	Inactive	Active	Inactive	Inactive	140	3
C300b	Inactive	Active	Inactive	Active	Inactive	Inactive	408	4
C316a	Inactive	Active	Inactive	Active	Inactive	Inactive	370	4
C334	Inactive	Active	Inactive	Active	Inactive	Inactive	300	3
C566a	Active	Active	Inactive	Active	Inactive	Inactive	400	4
C566b	Active	Active	Inactive	Active	Inactive	Inactive	400	4
C582a	Inactive	Active	Inactive	Active	Inactive	Inactive	408	4
C582b	Inactive	Active	Inactive	Active	Inactive	Inactive	408	4
C582c	Inactive	Active	Inactive	Active	Inactive	Inactive	408	4
C582d	Inactive	Active	Inactive	Active	Inactive	Inactive	185	3
C582e	Inactive	Active	Inactive	Active	Inactive	Inactive	185	3
C598a	Inactive	Active	Inactive	Active	Inactive	Inactive	185	3
C598b	Inactive	Active	Inactive	Active	Inactive	Inactive	370	4
C600a	Active	Active	Inactive	Active	Inactive	Active	300	3
C600b	Active	Active	Inactive	Active	Inactive	Active	300	3
C600c	Active	Active	Inactive	Active	Inactive	Active	300	3
exemestrane	Inactive	Active	Inactive	Active	Inactive	Inactive	292	5
letrozole	Inactive	Active	Inactive	Active	Inactive	Inactive	1463	4
neratinib	Inactive	Active	Active	Active	Inactive	Inactive	400	5

**Table 3:** The lowest EFEB (kcal/mol) for each photoproduct in the interaction with molecular targets aromatase, ER, FGFR1, EGFR, HER2, PR, and Beclin 1 was determined after 100 runs using the molecular docking simulation and predicted EFEB. The lowest predicted EFEB values from the identified photoproduct on each target are shown in **bold**. The overall lowest predicted EFEB value across all compounds on a target is indicated with an underline.

Molecule EFEB	Aromatase kcal/mol	ER kcal/mol	FGFR1 kcal/mol	EGFR kcal/mol	HER2 kcal/mol	PR kcal/mol	Beclin 1 kcal/mol
CPZ	−7.64	−8.49	−6.74	−8.42	−7.56	−8.69	−7.41
C178	−5.63	−5.27	−5.13	−6.29	−5.86	−5.42	−6.39
C284/PZ	−7.18	−7.96	−6.31	−7.61	−7.45	−7.90	−7.22
C300a	−7.20	−7.94	−6.97	−7.29	−7.21	−7.80	−7.46
C300b	−7.10	−7.71	−6.50	−7.98	−7.45	−8.19	−8.17
C316a	−7.08	−8.29	−6.74	−7.41	−7.53	−7.94	−6.95
C334	−7.69	−7.79	−7.18	−7.58	−7.55	−8.28	−7.39
C566a	−11.77	−9.00	−8.32	**−11.07**	−10.07	−8.62	−9.74
C566b	−11.17	**−9.26**	−8.63	−9.65	−9.95	−8.90	**−10.18**
C582a	−11.50	−7.92	−8.96	−9.91	−9.94	−8.21	−9.86
C582b	−11.27	−7.64	−8.85	−10.15	−8.88	−8.24	−10.07
C582c	−11.18	−9.05	−8.20	−10.78	−9.41	−8.74	−9.38
C582d	−11.29	−8.30	−9.19	−10.07	−8.68	**−9.55**	−9.33
C582e	−11.47	−7.17	**−9.81**	−9.53	−9.92	−9.04	−10.22
C598a	−10.99	−6.62	−9.40	−9.35	−8.17	−8.92	−9.25
C598b	−11.13	−8.25	−8.38	−10.27	−8.15	−8.36	−10.09
C600a	−10.90	−8.16	−8.04	−10.39	−10.04	−8.41	−9.40
C600b	**−11.81**	−9.03	−8.63	−10.26	−8.97	−8.45	−9.03
C600c	−11.48	−8.48	−8.63	−9.56	−9.28	−8.99	−9.91
exemestane	−9.13	−10.08	−8.28	−10.25	−8.51	−10.92	−8.98
letrozole	−8.73	−9.15	−8.79	−8.68	−8.62	−8.53	−7.87
neratinib	−11.83	−7.80	−8.65	−11.04	−9.41	−9.16	−8.31

**Table 4:** The grid point spacing, number of grid points, and coordinates of the central grid point of the maps for target proteins used in the molecular docking studies.

Target Protein	Grid Point Spacing (Angstroms)	Number of Grid Points (x,y,z)	Coordinates of Central Grid Point of Maps
Aromatase	0.375	102; 126; 112	83.659; 50.233; 46.605
HER-2	0.375	126; 108; 126	14.023; 21.179; 31.904
EGFR	0.375	126; 126; 126	0.410; 6.290; 18.351
FGFR1	0.375	82; 82; 126	42.690, 1.885, 3.118
Oestrogen receptor Alpha	0.375	98; 78; 126	26.173, −4.231, 69.321
Beclin 1	0.375	112; 98; 98	28.891, −3.953, 17.337

## Data Availability

Data are contained within the article.

## Supplementary Materials

The following supporting information can be downloaded at https://www.mdpi.com/article/10.3390/ijms26146668/s1.
