# Supplementary material for: Laser-Induced Dimeric Photoproducts of Chlorpromazine: LC-MS Identification and Molecular Docking Evidence of Enhanced Anticancer Potential
Source: Int J Mol Sci. 2025 Jul 11;26(14):6668. doi: 10.3390/ijms26146668 (PMC12294483; doi:10.3390/ijms26146668)
Supplement: Supplementary file 1 [file ijms-26-06668-s001.zip › ijms-3685175-supplementary.pdf]

1

Identification results of chlorpromazine degradation products.

Table S1 Identification results of chlorpromazine degradation products. Tr – retention time, Obs. Mass – recorded mass / charge (m/z), where z = 1, Theo. Mass – theoretical m/z of the precursor ion, Diff. – mass difference (accuracy), where Diff. = (Theo. Mass – Obs. Mass) / Theo. Mass × 106, CCV – Collision cell voltage, Ref. – reference.

**Compound**	**Molecular formula**	**Tr (min)**	**Detected ion**	**Obs. Mass (m/z)**	**Theo. Mass (m/z)**	**Diff. (ppm)**	**MS² fragments (m/z)**	**CCV (V)**	**SMILES**	**Ref.**
**CPZ**	$C_{17}H_{19}ClN_2S$	10.068	$[M+H]^+$	319.101784	319.102474	-2.16	274.05, 246.02, 239.08, 214.05, 86.1, 58.07	20	CN(C)CCCN1c2ccccc2Sc2ccc(Cl)cc12	[1]
**C178**	$C_{11}H_{18}N_2$	2.325	$[M+H]^+$	179.15478	179.153726	5.88	134.1, 106.06, 86.1, 77.04, 58.07	10	CN(C)CCCNc1ccccc1	
**C582a**	$C_{34}H_{38}N_4OS_2$	4.044	$[M+H]^+$	583.255764	583.255978	-0.37	497.17, 300.14, 256.07, 228.05, 215.05, 86.1, 58.07	20	CN(C)CCCN1c2ccccc2Sc2ccc(cc21)c1ccc2Sc3ccc(O)cc3N(CCCN(C)C)c2c1 **or** CN(C)CCCN1c2ccccc2Sc2ccc(cc21)c1ccc2N(CCCN(C)C)c3cc(O)ccc3Sc2c1 **or** CN(C)CCCN1c2ccccc2Sc2ccc(cc21)c1cc2Sc3ccccc3N(CCCN(C)C)c2cc1O **or** CN(C)CCCN1c2ccccc2S(=O)c2ccc(cc21)c1cc2N(CCCN(C)C)c3ccccc3Sc2cc1 **or** CN(C)CCCN1c2ccc(cc2S(=O)c2ccccc12)c1cc2N(CCCN(C)C)c3ccccc3Sc2cc1	
			$[M+2H]^{2+}$	292.129479	292.131353	-6.41	498.18, 300.13, 284.15, 255.07, 238.07, 215.04, 199.05, 86.1, 7.07, 58.07	15		
**C600a**	$C_{34}H_{37}ClN_4S_2$	4.271	$[M+H]^+$	601.220838	601.221543	-1.17	569.28, 517.15, 318.12, 273.03, 86.1, 58.07	20	CN(C)CCCN1c2ccccc2Sc2ccc(cc21)c1ccc2Sc3ccc(Cl)cc3N(CCCN(C)C)c2c1 **or** CN(C)CCCN1c2ccccc2Sc2ccc(cc21)c1ccc2N(CCCN(C)C)c3cc(Cl)ccc3Sc2c1 **or** CN(C)CCCN1c2ccccc2Sc2ccc(cc21)c1cc2Sc3ccccc3N(CCCN(C)C)c2cc1Cl	
			$[M+2H]^{2+}$	301.111943	301.1144095	-8.19	516.14, 318.1, 284.13, 272.05, 238.07, 212.05, 199.05, 86.1, 70.07, 58.07	10		
**C316**	$C_{17}H_{20}N_2O_2S$	5.489	$[M+H]^+$	317.131828	317.131276	1.74	300.13, 286.12, 272.08, 256.08, 242.07, 228.06, 215.04, 200.06, 167.08, 86.1, 58.07	20	CN(C)CCCN1c2ccccc2S(=O)c2ccc(O)cc12	[1]
**C300a**	$C_{17}H_{20}N_2OS$	5.943	$[M+H]^+$	301.135864	301.136361	-1.65	284.1, 270.13, 256.08, 239.09, 223.1, 199.05,	20	CN(C)CCCN1c2ccccc2S(=O)c2ccccc12	[1]

Compound	Molecular formula	Tr (min)	Detected ion	Obs. Mass (m/z)	Theo. Mass (m/z)	Diff. (ppm)	MS² fragments (m/z)	CCV (V)	SMILES	Ref.
							180.09, 133.06, 86.1, 72.08, 58.07			
**C598a**	$C_{34}H_{38}N_4O_2S_2$	6.851	$[M+H]^+$	599.247216	599.250344	-5.22	583.27, 555.18, 529.15, 513.14, 498.23, 452.13, 424.1, 414.09, 392.19, 304.06, 271.02, 250.1, 86.1, 58.07	20	CN(C)CCCN1c2ccccc2S(=O)c2ccc(cc21)c1cc2N(CCCN(C)C)c3ccccc3S(=O)c2cc1 **or** CN(C)CCCN1c2cc(O)ccc2S(=O)c2ccc(cc21)c1cc2N(CCCN(C)C)c3ccccc3Sc2cc1	
			$[M+2H]^{2+}$	300.126619	300.1288105	-7.30	514.18, 496.18, 468.24, 453.15, 339.11, 86.1, 72.08, 58.07	10		
**C334**	$C_{17}H_{19}ClN_2OS$	7.046	$[M+H]^+$	335.097777	335.097389	1.16	318.1, 304.08, 290.05, 273.04, 255.08, 246.02, 232.01, 225.07, 214.05, 86.1, 72.08, 58.06	20	CN(C)CCCN1c2ccccc2S(=O)c2ccc(Cl)cc12	[1,2]
**C582b**	$C_{34}H_{38}N_4OS_2$	7.46	$[M+H]^+$	583.254673	583.255978	-2.24	566.21, 539.16, 510.16, 497.2, 460.17, 430.04, 420.14, 389.17, 314.08, 260.03, 152.07, 86.1, 72.09, 58.07	20	CN(C)CCCN1c2ccccc2Sc2ccc(cc21)c1ccc2Sc3ccc(O)cc3N(CCCN(C)C)c2c1 **or** CN(C)CCCN1c2ccccc2Sc2ccc(cc21)c1ccc2N(CCCN(C)C)c3cc(O)ccc3Sc2c1 **or** CN(C)CCCN1c2ccccc2Sc2ccc(cc21)c1cc2Sc3ccccc3N(CCCN(C)C)c2cc1O **or** CN(C)CCCN1c2ccccc2S(=O)c2ccc(cc21)c1cc2N(CCCN(C)C)c3ccccc3Sc2cc1 **or** CN(C)CCCN1c2ccc(cc2S(=O)c2ccccc12)c1cc2N(CCCN(C)C)c3ccccc3Sc2cc1	
			$[M+2H]^{2+}$	292.129329	292.131353	-6.93	514.17, 498.18, 453.15, 425.07, 412.06, 158.97, 86.1, 70.07, 58.07	10		
**C300b**	$C_{17}H_{20}N_2OS$	7.968	$[M+H]^+$	301.134866	301.136361	-4.96	256.08, 228.05, 215.04, 86.1, 58.07	15	CN(C)CCCN1c2ccccc2Sc2ccc(O)cc12	[1,2]
**C582c**	$C_{34}H_{38}N_4OS_2$	8.357	$[M+H]^+$	583.255821	583.255978	-0.27	86.1, 58.07	20	CN(C)CCCN1c2ccccc2Sc2ccc(cc21)c1ccc2Sc3ccc(O)cc3N(CCCN(C)C)c2c1 **or** CN(C)CCCN1c2ccccc2Sc2ccc(cc21)c1ccc2N(CCCN(C)C)c3cc(O)ccc3Sc2c1 **or**	
			$[M+2H]^{2+}$	292.128309	292.131353	-10.42	498.17, 453.12, 412.08, 86.1, 58.07	10		

Compound	Molecular formula	Tr (min)	Detected ion	Obs. Mass (m/z)	Theo. Mass (m/z)	Diff. (ppm)	MS² fragments (m/z)	CCV (V)	SMILES	Ref.
									CN(C)CCCN1c2ccccc2Sc2ccc(cc21)c1cc2Sc3ccccc3N(CCCN(C)C)c2cc1O **or** CN(C)CCCN1c2ccccc2S(=O)c2ccc(cc21)c1cc2N(CCCN(C)C)c3ccccc3Sc2cc1 **or** CN(C)CCCN1c2ccc(cc2S(=O)c2ccccc12)c1cc2N(CCCN(C)C)c3ccccc3Sc2cc1	
**C582d**	$C_{34}H_{38}N_4OS_2$	8.663	$[M+H]^+$	583.255908	583.255978	-0.12	86.1, 58.7	20	CN(C)CCCN1c2ccccc2Sc2ccc(cc21)c1ccc2Sc3ccc(O)cc3N(CCCN(C)C)c2c1 **or** CN(C)CCCN1c2ccccc2Sc2ccc(cc21)c1ccc2N(CCCN(C)C)c3cc(O)ccc3Sc2c1 **or** CN(C)CCCN1c2ccccc2Sc2ccc(cc21)c1cc2Sc3ccccc3N(CCCN(C)C)c2cc1O **or** CN(C)CCCN1c2ccccc2S(=O)c2ccc(cc21)c1cc2N(CCCN(C)C)c3ccccc3Sc2cc1 **or** CN(C)CCCN1c2ccc(cc2S(=O)c2ccccc12)c1cc2N(CCCN(C)C)c3ccccc3Sc2cc1	
			$[M+2H]^{2+}$	292.129171	292.131353	-7.47	498.17, 453.11, 425.09, 412.08, 86.1, 58.07	10		
**C598d**	$C_{34}H_{38}N_4O_2S_2$	9.053	$[M+H]^+$	599.246729	599.250344	-6.03	583.24, 554.18, 537.21, 526.16, 513.18, 301.13, 285.14, 255.06, 199.03, 86.1, 58.07	20	CN(C)CCCN1c2ccccc2S(=O)c2ccc(cc21)c1cc2N(CCCN(C)C)c3ccccc3S(=O)c2cc1 **or** CN(C)CCCN1c2cc(O)ccc2S(=O)c2ccc(cc21)c1cc2N(CCCN(C)C)c3ccccc3Sc2cc1	
			$[M+2H]^{2+}$	300.131618	300.1290845	8.44	514.17, 469.11, 441.08, 428.07, 86.1, 58.07	10		
**C284/PZ**	$C_{17}H_{20}N_2S$	9.174	$[M+H]^+$	285.141288	285.141995	-2.48	240.09, 212.06, 199.05, 180.08, 86.1, 58.07	20	CN(C)CCCN1c2ccccc2Sc2ccccc12	[1,2]
**C566a**	$C_{34}H_{38}N_4S_2$	9.42	$[M+H]^+$	567.261565	567.261064	0.88	86.1, 58.07	20	CN(C)CCCN1c2ccccc2Sc2ccc(cc21)c1cc2N(CCCN(C)C)c3ccccc3Sc2cc1 **or** CN(C)CCCN1c2ccccc2Sc2ccc(cc21)c1ccc2N(CCCN(C)C)c3ccccc3Sc2c1	
			$[M+2H]^{2+}$	284.132246	284.1338955	-5.81	482.18, 437.12, 409.09, 86.1, 58.07	15		
**C600b**	$C_{34}H_{37}ClN_4S_2$	9.563	$[M+H]^+$	601.221751	601.221543	0.35	567.27, 558.16, 543.15, 530.17, 86.1, 58.07	20	CN(C)CCCN1c2ccccc2Sc2ccc(cc21)c1ccc2Sc3ccc(Cl)cc3N(CCCN(C)C)c2c1	

**Compound**	**Molecular formula**	**Tr (min)**	**Detected ion**	**Obs. Mass (m/z)**	**Theo. Mass (m/z)**	**Diff. (ppm)**	**MS² fragments (m/z)**	**CCV (V)**	**SMILES**	**Ref.**
			$[M+2H]^{2+}$	301.112351	301.1144095	-6.84	516.14, 471.08, 443.04, 86.1, 58.07	10	**or**<br>CN(C)CCCN1c2ccccc2Sc2ccc(cc21)c1ccc2N(CCCN(C)C)c3cc(Cl)ccc3Sc2c1<br>**or**<br>CN(C)CCCN1c2ccccc2Sc2ccc(cc21)c1cc2Sc3ccccc3N(CCCN(C)C)c2cc1Cl	
**C582e**	$C_{34}H_{38}N_4OS_2$	9.665	$[M+H]^+$	583.253463	583.255978	-4.31	512.2, 465.1, 452.05, 427.09, 86.1, 58.07	20	CN(C)CCCN1c2ccccc2Sc2ccc(cc21)c1ccc2Sc3ccc(O)cc3N(CCCN(C)C)c2c1<br>**or**<br>CN(C)CCCN1c2ccccc2Sc2ccc(cc21)c1ccc2N(CCCN(C)C)c3cc(O)ccc3Sc2c1<br>**or**<br>CN(C)CCCN1c2ccccc2Sc2ccc(cc21)c1cc2Sc3ccccc3N(CCCN(C)C)c2cc1O<br>**or**<br>CN(C)CCCN1c2ccccc2S(=O)c2ccc(cc21)c1cc2N(CCCN(C)C)c3ccccc3Sc2cc1<br>**or**<br>CN(C)CCCN1c2ccc(cc2S(=O)c2ccccc12)c1cc2N(CCCN(C)C)c3ccccc3Sc2cc1	
			$[M+2H]^{2+}$	292.129544	292.131353	-6.19	498.18, 453.12, 425.09, 412.07, 86.1, 58.07	10		
**C566b**	$C_{34}H_{38}N_4S_2$	9.754	$[M+H]^+$	567.259984	567.261064	-1.90	522.18, 494.15, 482.15, 438.07, 159, 86.1, 72.09, 58.07	20	CN(C)CCCN1c2ccccc2Sc2ccc(cc21)c1cc2N(CCCN(C)C)c3ccccc3Sc2cc1<br>**or**<br>CN(C)CCCN1c2ccccc2Sc2ccc(cc21)c1ccc2N(CCCN(C)C)c3ccccc3Sc2c1	
			$[M+2H]^{2+}$	284.131283	284.1338955	-9.19	482.18, 437.12, 409.09, 396.08, 86.1, 58.07	15		
**C600c**	$C_{34}H_{37}ClN_4S_2$	9.988	$[M+H]^+$	601.22056	601.221543	-1.64	86.1, 58.07	20	CN(C)CCCN1c2ccccc2Sc2ccc(cc21)c1ccc2Sc3ccc(Cl)cc3N(CCCN(C)C)c2c1<br>**or**<br>CN(C)CCCN1c2ccccc2Sc2ccc(cc21)c1ccc2N(CCCN(C)C)c3cc(Cl)ccc3Sc2c1<br>**or**<br>CN(C)CCCN1c2ccccc2Sc2ccc(cc21)c1cc2Sc3ccccc3N(CCCN(C)C)c2cc1Cl	
			$[M+2H]^{2+}$	301.111346	301.1144095	-10.17	516.14, 471.08, 443.05, 86.1, 58.07	10		

SMILES codes and the 2D structures of CPZ and its identified photoproducts using the HPLC-MS analyses.

Table S2. SMILES code of the compounds and 2D chemical structure of CPZ and its identified photoproducts.

Compound	SMILES code	2D chemical structure
CPZ	CN(C)CCCN1c2ccccc2Sc2ccc(Cl)cc12	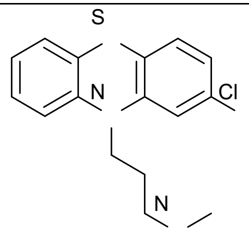
C178	CN(C)CCCNc1ccccc1	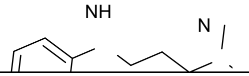
C284/PZ	CN(C)CCCN1c2ccccc2Sc2ccccc12	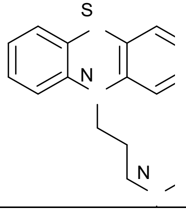
C300a	CN(C)CCCN1c2ccccc2S(=O)c2ccccc12	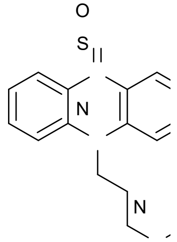
C300b	CN(C)CCCN1c2ccccc2Sc2ccc(O)cc12	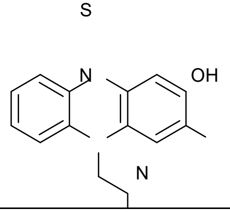
C316a	CN(C)CCCN1c2ccccc2S(=O)c2ccc(O)cc12	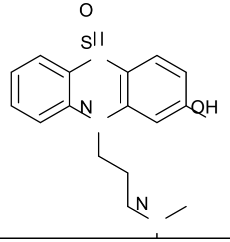

C334	CN(C)CCCN1c2ccccc2S(=O)c2ccc(Cl)cc12	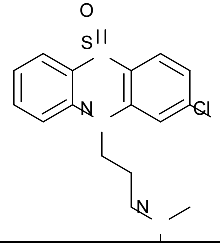
C566a	CN(C)CCCN1c2ccccc2Sc2ccc(cc21)c1cc2N(CCCN(C)C)c3ccccc3Sc2cc1	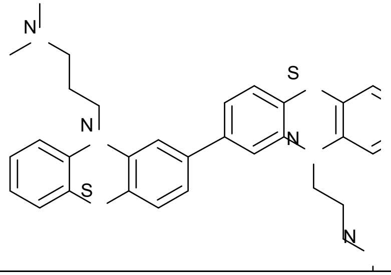
C566b	CN(C)CCCN1c2ccccc2Sc2ccc(cc21)c1ccc2N(CCCN(C)C)c3ccccc3Sc2c1	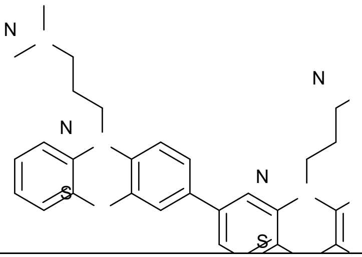
C582a	CN(C)CCCN1c2ccccc2Sc2ccc(cc21)c1ccc2Sc3ccc(O)cc3N(CCCN(C)C)c2c1	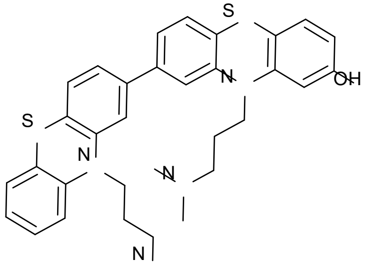

C582b	CN(C)CCCN1c2ccccc2Sc2ccc(cc21)c1ccc2N(CCCN(C)C)c3cc(O)ccc3Sc2c1	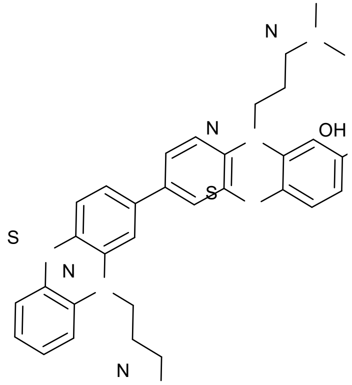
C582c	CN(C)CCCN1c2ccccc2Sc2ccc(cc21)c1cc2Sc3ccccc3N(CCCN(C)C)c2cc1O	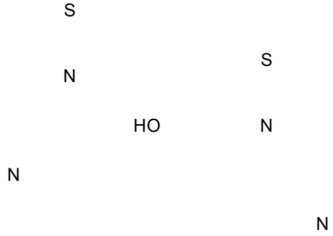
C582d	CN(C)CCCN1c2ccccc2S(=O)c2ccc(cc21)c1cc2N(CCCN(C)C)c3ccccc3Sc2cc1	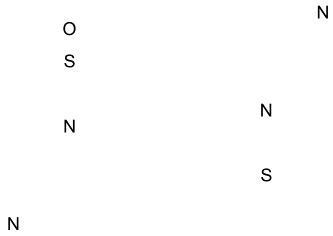
C582e	CN(C)CCCN1c2ccc(cc2S(=O)c2ccccc12)c1cc2N(CCCN(C)C)c3ccccc3Sc2cc1	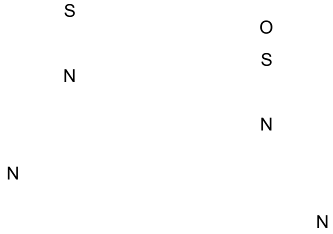

C598a	CN(C)CCCN1c2ccccc2S(=O)c2ccc(cc21)c1cc2N(CCCN(C)C)c3ccccc3S(=O)c2cc1	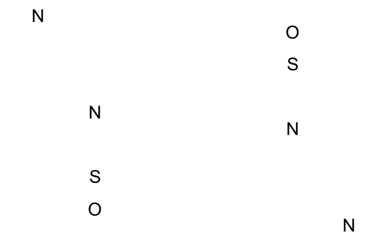
C598b	CN(C)CCCN1c2cc(O)ccc2S(=O)c2ccc(cc21)c1cc2N(CCCN(C)C)c3ccccc3Sc2cc1	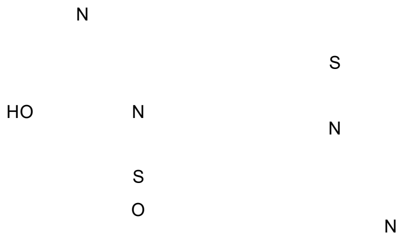
C600a	CN(C)CCCN1c2ccccc2Sc2ccc(cc21)c1ccc2Sc3ccc(Cl)cc3N(CCCN(C)C)c2c1	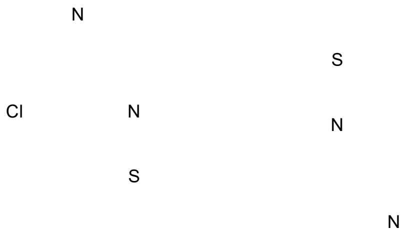
C600b	CN(C)CCCN1c2ccccc2Sc2ccc(cc21)c1ccc2N(CCCN(C)C)c3cc(Cl)ccc3Sc2c1	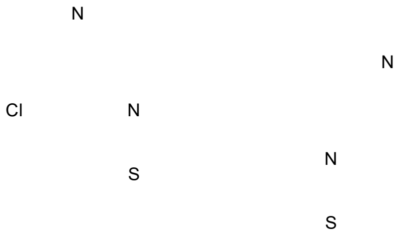

C600c	CN(C)CCCN1c2ccccc2Sc2ccc(cc21)c1cc2Sc3ccccc3N(CCCN(C)C)c2cc1Cl	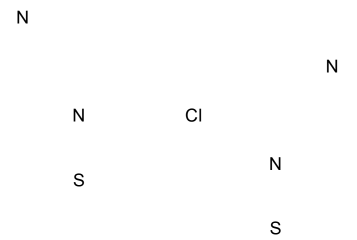

SwissADME results of CPZ photoproducts compared with CPZ

Table S3. SwissADME results for CPZ and its photoproducts. Parameters include drug-likeness violations (Lipinski, Ghose, Veber, Egan, and Muegge rules), bioavailability score, PAINS and Brenk alerts, lead-likeness violations, and synthetic accessibility.

Molecule	Lipinski	Ghose	Veber	Egan	Muegge	Bioavailability	PAINS	Brenk	Leadlikeness	Synthetic accessibility
CPZ	1	0	0	0	1	0.55	0	0	1	3.12
C178	0	0	0	0	1	0.55	0	0	1	1.25
C284/PZ	0	0	0	0	0	0.55	1	0	1	3.11
C300a	0	0	0	0	0	0.55	1	0	0	3.29
C300b	0	0	0	0	0	0.55	0	0	1	3.13
C316a	0	0	0	0	0	0.55	0	0	0	3.75
C334	0	0	0	0	0	0.55	0	0	0	3.74
C566a	2	4	0	1	1	0.17	1	0	3	4.51
C566b	2	4	0	1	1	0.17	1	0	3	4.62
C582a	2	4	0	1	1	0.17	1	0	3	4.55
C582b	2	4	0	1	1	0.17	1	0	3	4.65
C582c	2	4	0	1	1	0.17	1	0	3	4.67
C582d	2	4	0	1	1	0.17	1	0	3	5.13
C582e	2	4	0	1	1	0.17	1	0	3	5.25
C598a	2	4	0	1	1	0.17	1	0	3	5.44
C598b	2	4	0	1	1	0.17	1	0	3	5.16
C600a	2	4	0	1	2	0.17	1	0	3	4.53
C600b	2	4	0	1	2	0.17	1	0	3	4.63
C600c	2	4	0	1	2	0.17	1	0	3	4.68

ADME results of CPZ photoproducts compared with CPZ.

Table S4 ADME results of CPZ photoproducts compared with CPZ. Human Oral Bioavailability (HOB); Human Intestinal Absorption (HIA); Fraction Unbound (Human); Plasma Protein Binding Predictions, Steady State Volume of Distribution (VD), Organic Cation Transporter 2 (OCT2), Half-Life of Drug Predictions; Maximum Tolerated Dose Predictions

	CPZ	C178	C284 /PZ	C300a	C300b	C316a	C334	C566a	C566b	C582a	C582b	C582 a	C582 b	C582 c	C598 d	C598e	C600 a	C600b	C600c
HOB 20% Predictions	Yes	Yes	Yes	Yes	Yes	Yes	Yes	Yes	Yes	Yes	Yes	No	Yes	Yes	Yes	Yes	Yes	Yes	Yes
HOB 20% Probability	0.84	0.86	0.79	0.83	0.70	0.77	0.87	0.62	0.62	0.52	0.50	0.47	0.67	0.64	0.73	0.60	0.68	0.69	0.69
HIA Predictions	Abs.	Abs.	Abs.	Abs.	Abs.	Abs.	Abs.	Abs.	Abs.	Abs.	Abs.	Abs.	Abs.	Abs.	Abs.	Abs.	Abs.	Abs.	Abs.
HIA Probability	0.99	0.93	0.98	0.99	0.99	0.98	0.99	0.90	0.918	0.86	0.89	0.91	0.95	0.91	0.91	0.896	0.96	0.96	0.97
Fraction Unbound	1.96	0.42	1.82	1.21	0.81	0.7	1.49	2.12	2.13	1.55	1.54	1.64	1.91	1.91	1.87	1.49	2.03	2.04	2.16
Plasma Protein Binding	44.2	-4.02	27.4	29.1	48.1	39.9	42.9	56.8	57.49	73.1	73.5	74.7	64.6	66.2	63.7	75.48	62.0	62.5	62.1
VD	6.61	6.18	8.14	5.03	4.78	2.86	4.44	6.56	6.49	4.37	4.36	4.67	5.37	4.81	4.18	3.58	5.95	5.89	5.92
Clearance	10.93	10.56	13.83	10.83	9.5	7.59	8.87	11.7	11.67	10.74	10.47	9.64	11.31	11.09	10.44	10.71	10.95	10.94	10.8
OCT 2 Ingibitor	Yes	No	Yes	Yes	Yes	Yes	Yes	No	No	No	No	No	No	No	No	No	No	No	No
OCT 2 Probability	0.721	0.461	0.765	0.575	0.626	0.57	0.552	0.304	0.314	0.363	0.376	0.385	0.233	0.216	0.279	0.321	0.23	0.237	0.221
Half-Life of Drug	< 3hs	< 3hs	< 3hs	< 3hs	< 3hs	< 3hs	< 3hs	< 3hs	< 3hs	< 3hs	< 3hs	< 3hs	< 3hs	< 3hs	< 3hs	< 3hs	< 3hs	< 3hs	< 3hs
Max. Tolerated Dose	0.87	0.59	0.79	0.63	0.41	0.31	0.64	0.82	0.83	0.63	0.66	0.6	0.64	0.34	0.56	0.56	0.7	0.7	0.74
hERG Blockers	Tox. (HC)	Safe (HC)	Tox. (HC)	Tox. (MC)	Tox. (MC)	Tox. (MC)	Tox. (HC)	Tox. (HC)	Tox. (HC)	Tox. (MC)	Tox. (HC)	Tox. (HC)	Tox. (HC)	Tox. (HC)	Tox. (MC)	Tox. (MC)	Tox. (HC)	Tox. (HC)	Tox. (HC)
NR-AhR	Safe (HC)	Safe (HC)	Safe (HC)	Safe (HC)	Safe (LC)	Safe (MC)	Safe (HC)	Safe (HC)	Safe (HC)	Safe (HC)	Safe (HC)	Safe (HC)	Safe (HC)	Safe (HC)	Safe (HC)	Safe (HC)	Safe (HC)	Safe (HC)	Safe (HC)
NR-AR-LBD	Safe (HC)	Safe (HC)	Safe (HC)	Safe (HC)	Safe (HC)	Safe (HC)	Safe (HC)	Safe (HC)	Safe (HC)	Safe (HC)	Safe (HC)	Safe (HC)	Safe (HC)	Safe (HC)	Safe (HC)	Safe (HC)	Safe (HC)	Safe (HC)	Safe (HC)

NR-Aromatase	Safe (HC)	Safe (HC)	Safe (HC)	Safe (HC)	Safe (HC)	Safe (HC)	Safe (HC)	Tox. (HC)	Tox. (HC)	Tox. (HC)	Tox. (HC)	Safe (MC)	Safe (HC)	Safe (HC)	Safe (HC)	Safe (MC)	Tox. (HC)	Tox. (HC)	Tox. (HC)
NR-ER-LBD	Safe (HC)	Safe (HC)	Safe (HC)	Safe (HC)	Safe (HC)	Safe (HC)	Safe (HC)	Safe (HC)	Safe (HC)	Safe (HC)	Safe (HC)	Safe (HC)	Safe (HC)	Safe (HC)	Safe (HC)	Safe (HC)	Safe (HC)	Safe (HC)	Safe (HC)
NR-PPAR-gamma	Safe (HC)	Safe (HC)	Safe (HC)	Safe (HC)	Safe (HC)	Safe (HC)	Safe (HC)	Safe (HC)	Safe (HC)	Safe (HC)	Safe (HC)	Safe (HC)	Safe (HC)	Safe (HC)	Safe (HC)	Safe (HC)	Safe (HC)	Safe (HC)	Safe (HC)
SR-p53	Tox. (LC)	Safe (HC)	Safe (HC)	Safe (HC)	Safe (HC)	Safe (HC)	Safe (MC)	Tox. (HC)	Tox. (HC)	Tox. (HC)	Tox. (HC)	Tox. (HC)	Tox. (MC)	Tox. (HC)	Safe (LC)	Tox. (MC)	Tox. (HC)	Tox. (HC)	Tox. (HC)

Extracted ion current (EIC) chromatograms

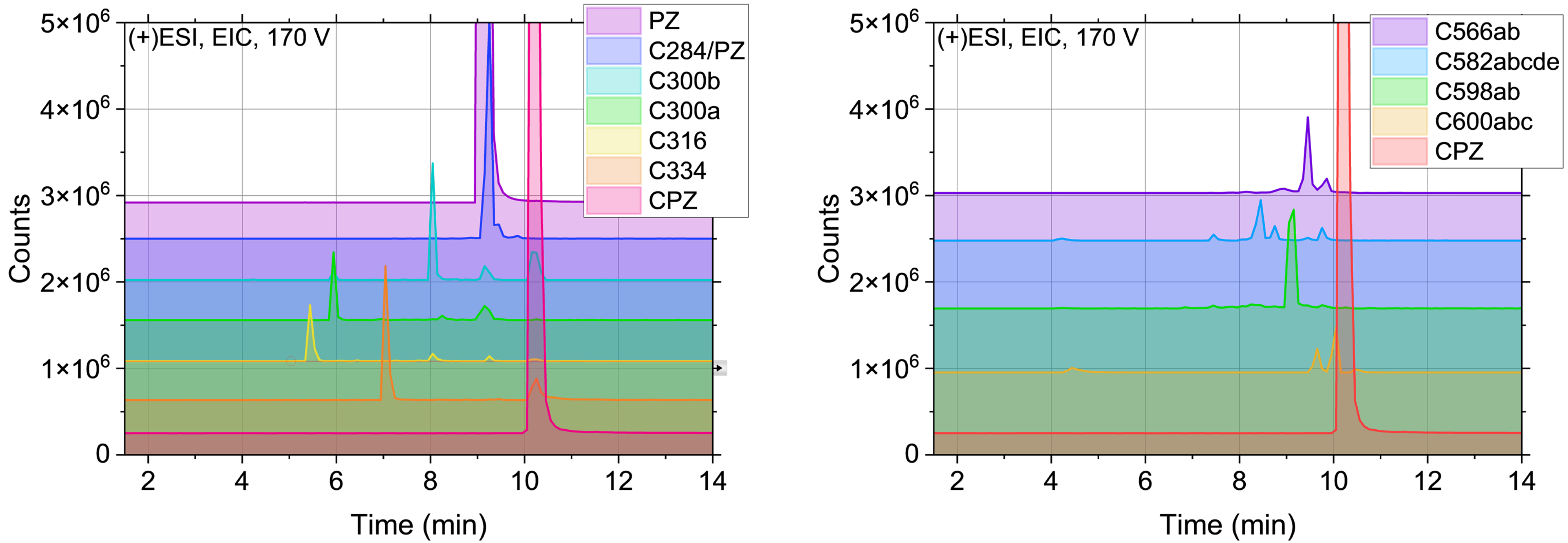

A. B.

Figure S1 Extracted ion current (EIC) chromatograms for **A.** the 20 mg/L standard solutions of PZ and CPZ and the photoproducts at the treatment time where the maximum abundance was recorded (5 min for C284 and C300b; 60 min for C300a, C316 and C334) and **B.** dimeric photoproducts at the treatment time where the maximum abundance was recorded (5 min for C600abc; 40 min for C582abcde and C598ab, 60 min for C566ab). PZ – promazine, CPZ – Chlorpromazine.

Mass spectra (MS) of C566a

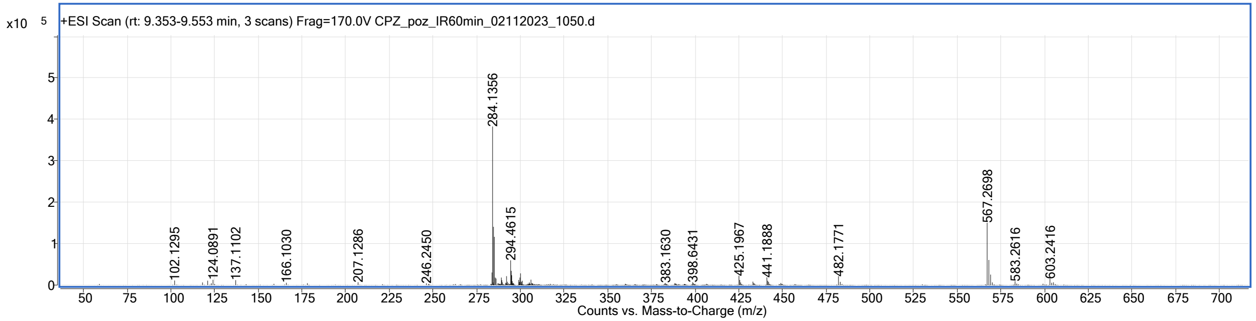

A.

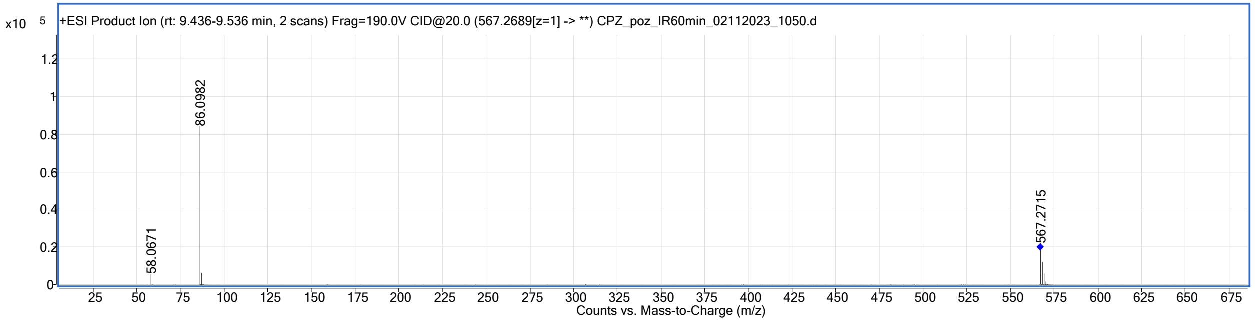

B.

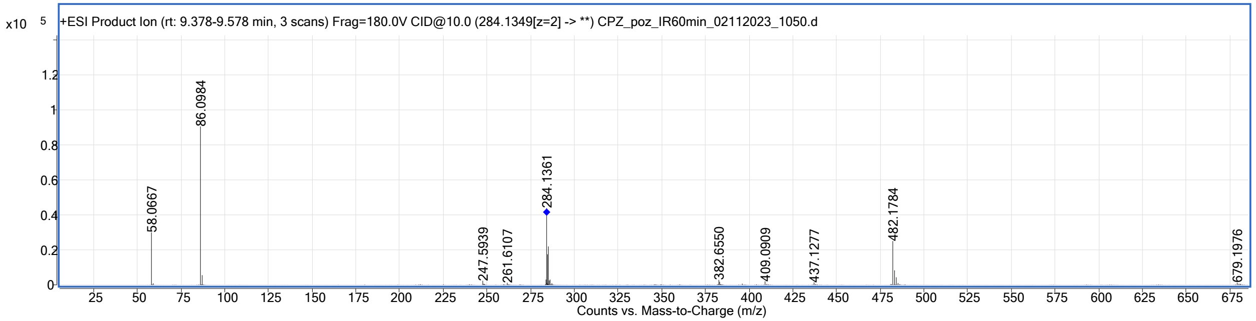

C.

Figure S2 Mass spectra (MS) of C566a: **A.** Full Scan MS; **B.** MS^2 for the $[M+H]^+$ precursor ion; **C.** MS^2 for the $[M+2H]^{2+}$ precursor ion.

Mass spectra (MS) of C582c

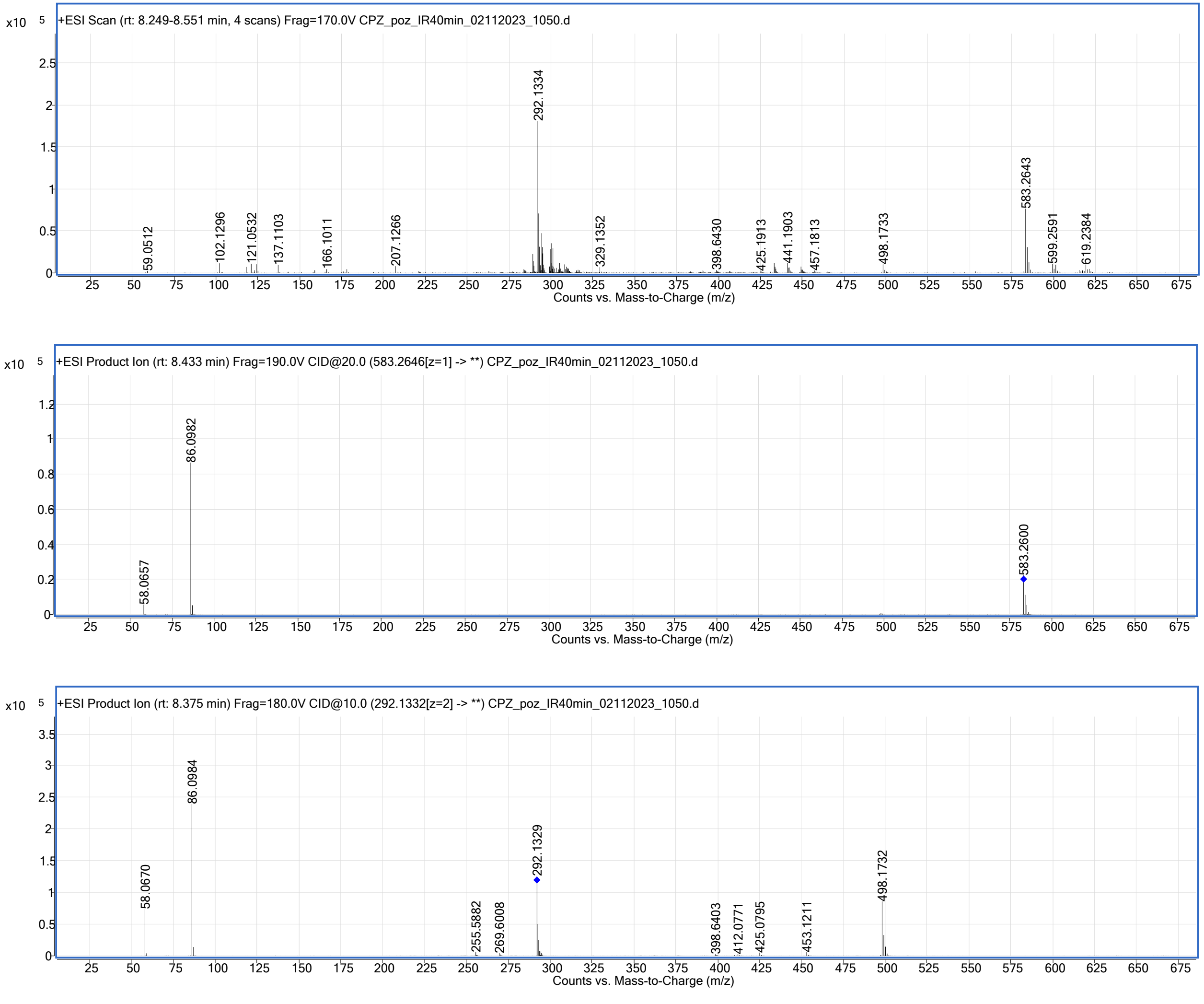

Figure S3 Mass spectra (MS) of C582c: **A.** Full Scan MS; **B.** MS^2 for the $[M+H]^+$ precursor ion; **C.** MS^2 for the $[M+2H]^{2+}$ precursor ion.

Mass spectra (MS) of C598b

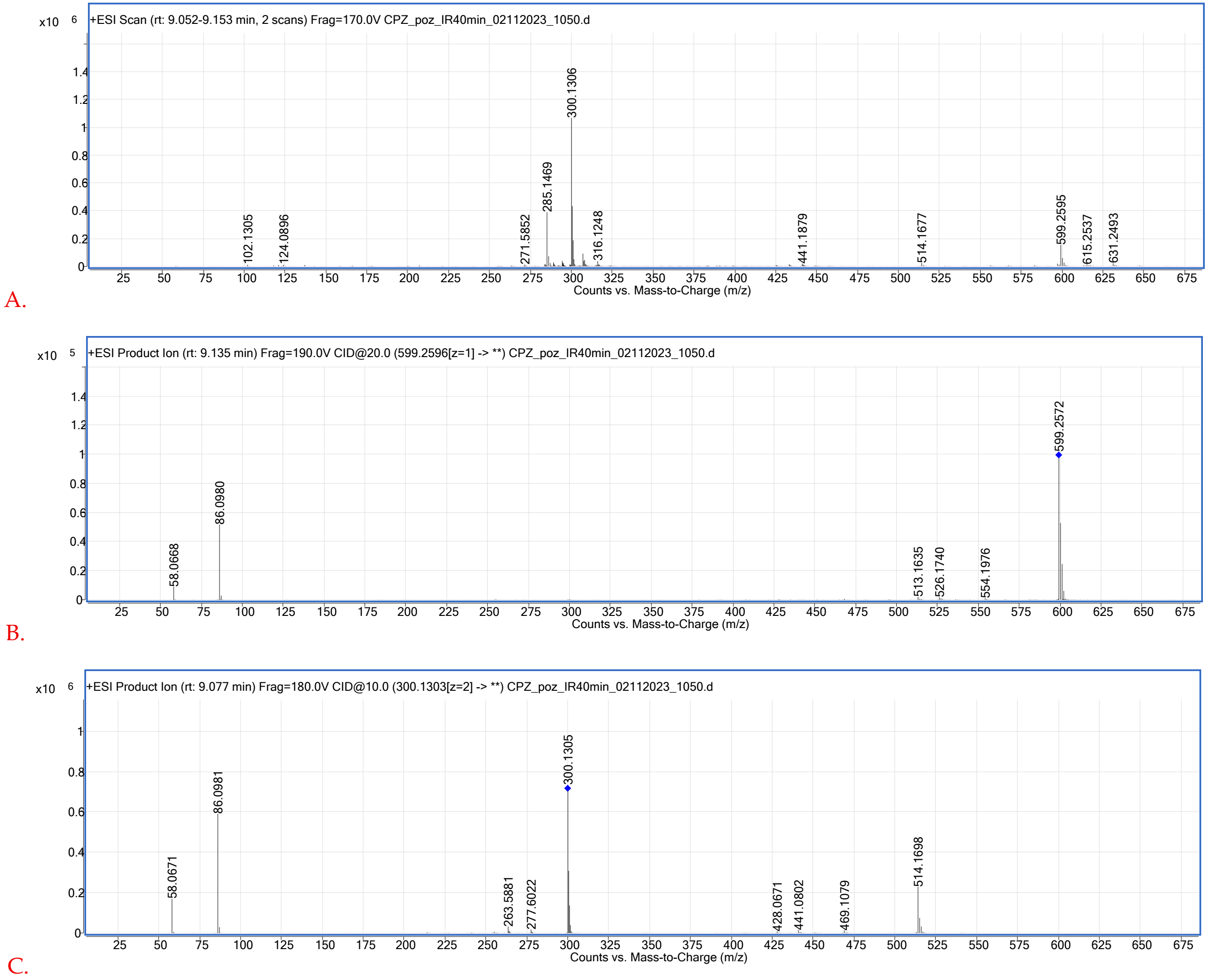

Figure S4 Mass spectra (MS) of C598b: **A.** Full Scan MS; **B.** MS^2 for the $[M+H]^+$ precursor ion; **C.** MS^2 for the $[M+2H]^{2+}$ precursor ion.

Mass spectra (MS) of C600c

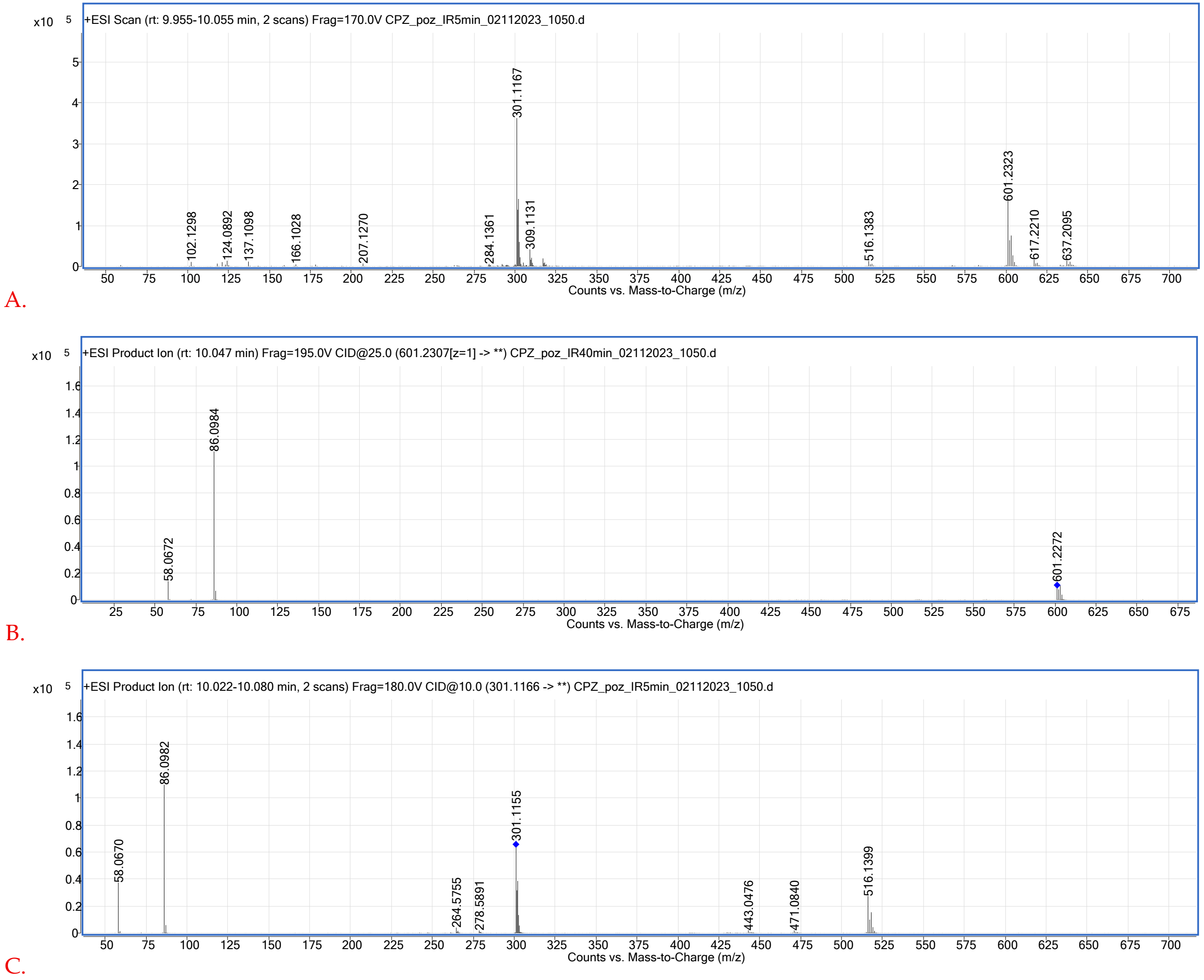

Figure S5 Mass spectra (MS) of C600c: **A.** Full Scan MS; **B.** MS^2 for the $[M+H]^+$ precursor ion; **C.** MS^2 for the $[M+2H]^{2+}$ precursor ion.
